# PERCEIVED ROLES IN MEDICATION SAFETY: PATIENTS' PERSPECTIVES

**DOI:** 10.1093/geroni/igac059.2577

**Published:** 2022-12-20

**Authors:** Fatoumata Jallow, Elisa Stehling, Zara Sajwani, Kathryn Daniel, Yan Xiao, Kimberly Fulda, Anna Espinoza

**Affiliations:** University of Texas at Arlington, Arlington, Texas, United States; University of Texas at Arlington, Arlington, Texas, United States; Children's Health, Dallas, Texas, United States; University of Texas at Arlington, Arlington, Texas, United States; University of Texas at Arlington, Arlington, Texas, United States; University of North Texas Health Science Center, Fort Worth, Texas, United States; University of North Texas Health Science Center, Fort Worth, Texas, United States

## Abstract

Community-dwelling older adults are vulnerable to medication safety-related harms. Prevention of medication-related harms in the outpatient setting starts with thorough and thoughtful medication reconciliation at each patient encounter. Comprehensive medication reconciliation is challenging for prescribers to provide in busy time-pressured practices. Older adults currently taking five or more daily prescription medications were recruited for this qualitative study. From the participants’ perspective, we explored the role of the prescriber, pharmacist, and patient in medication safety. During the COVID-19 pandemic, interviews were conducted from October 2020 to June 2021. Results from these interviews suggest that older adults recognized their role in medication safety supersedes just taking the pills as prescribed. Older adults understand that they must play an essential role in the coproduction of quality health services. Subthemes that emerged from the patient’s perceived role were “taking fewer medications,” “locking them up,” “keeping appointments,” and “reading the label.” Pharmacists were expected to inform participants of any changes in their medications, such as the color, shape, or dosage, and ensure no drug interactions. Primary care providers are expected to coordinate care between all specialists treating their patients and any medication prescribed by those specialists. There was a high level of trust in the provider’s knowledge, skill, and experience, along with a low level of patient engagement in decision-making around deprescribing. Among older adults, self-perceptions of their role in medication safety varied widely. Educating prescribers and pharmacists about the role expectations of this vulnerable population can help improve medication safety.

